# ALASKA NATIVES AND GENERATIVITY: BENEFITS AND CHALLENGES OF GENERATIVITY IN A RAPIDLY CHANGING ARCTIC

**DOI:** 10.1093/geroni/igac059.429

**Published:** 2022-12-20

**Authors:** Jordan Lewis

**Affiliations:** University of Minnesota Medical School, Duluth, Minnesota, United States

## Abstract

Generativity is not often associated with successful aging, but aligns with the cultural values and practices of Alaska Native Elders. This study explored the concept of generativity, why elders share their knowledge, how it makes them feel, and what they want everyone to know. This study interviewed 108 Alaska Native Elders across Alaska, ages ranging from 48 - 93 years old, and willing to share their knowledge of successful aging. Employing content analysis, we explored themes related to each research question on generativity. Elders felt motivated, happy, and good about sharing their knowledge, but only when others listened. Their motivations were to teach the real history of Alaska, leave a community or family legacy, and help youth engage in healthy behaviors. Knowing cultural values, your history, practical skills, and how to listen to your Elders were things they want to share with youth and fellow Elders. Participants also shared the challenges to being generative, including technology, lack of interest by others, and age differences in experiences and knowledge sought. We learned the importance of addressing the generative mismatch between generations and to improve quality of life for Elders and help youth age successfully, we need to create culturally meaningful activities to bring all generations together. Programs to teach youth to be healthy and develop a healthy identity through cultural practices and values, and provide Elders outlets to share and improve their quality of life will ensure cultural survival and foster and strengthen the cultural practice of Indigenous cultural generativity.

